# ID 170 - Desenvolvimento de uma Tecnologia Educacional sobre Protocolo de Uso

**DOI:** 10.5327/2237-9622.2025.v34s1.170

**Published:** 2025-11-25

**Authors:** Caroline Engster da Silva, Anna Gabriela Cavalcanti Arais, Caroline Engster da Silva, Airton Tetelbom Stein, Rita Catalina Aquino Caregnato

**Keywords:** tecnologias em saúde, protocolo, prática clínica baseada em evidências, ensino

## Abstract

**Introdução:**

No Brasil, a Comissão Nacional de Incorporação de Tecnologias no Sistema Único de Saúde (Conitec), dispõe sobre a assistência terapêutica e a incorporação de tecnologia em saúde no âmbito do SUS, definindo diferentes tipos de nomenclaturas para denominar os Guidelines, sendo o Protocolo de Uso uma das mais desconhecidas pelos profissionais da saúde. O objetivo é desenvolver uma tecnologia educacional que divulgue o Protocolo de Uso para os profissionais da saúde no Brasil.

**Método:**

Trata-se de uma pesquisa desenvolvida pelo método Delphi Modificado, com 12 participantes na primeira rodada e 10 na segunda rodada. O campo de ação foram os Núcleos de Avaliação de Tecnologias em Saúde (Nats) cadastrados na Rede Brasileira de Tecnologia em Saúde. Amostra não probabilística com seleção dos profissionais participantes dos Nats, seguida da técnica "bola-de-neve" com experts que atuam na área de Avaliação de Tecnologias em Saúde (ATS). Coleta de dados ocorreu em duas rodadas: elaboração de um formulário com 12 perguntas abertas que conduziram as entrevistas individuais face a face com experts realizadas on-line, com posterior análise de conteúdo de Bardin; e elaboração de um formulário com oito questões quantitativas, fundamentado nas respostas da primeira rodada, alcançando índice de consenso superior a 80% entre os experts, com posterior devolutiva dos resultados aos participantes.

**Resultados:**

Os achados de cada rodada foram fundamentais para a obtenção das recomendações dos experts para o Protocolo de Uso. Apesar de 83,3% dos especialistas desconhecerem a nomenclatura, após a contextualização, concordaram na importância e relevância desse documento. Na primeira rodada, houve índice de consenso superior a 80%, em aspectos relativos à criação e à utilização do Protocolo de Uso como a importância para o gerenciamento das instituições e a utilização do mesmo percurso metodológico usado na criação dos Protocolos Assistenciais. Na segunda rodada, obteve-se índice de consenso médio de 88,75% nas questões quantitativas relativas a fatores como garantia de legitimidade do documento; viabilidade e modelo de um protocolo para utilização interdisciplinar e a abordagem com foco nos processos e procedimentos e não na categoria profissional. Esses achados permitiram a editoração de um e-book intitulado .

**Conclusão:**

A pesquisa permitiu conhecer a opinião dos especialistas e aprofundar o conceito sobre o Protocolo de Uso, servindo de fundamentação para a construção de uma tecnologia educacional direcionada aos profissionais de saúde. O Protocolo de Uso é fundamentado em evidências científicas para prestar uma assistência qualificada e segura ao paciente dependente de dispositivos tecnológicos.

